# Differences in motor response to stability perturbations limit fall-resisting skill transfer

**DOI:** 10.1038/s41598-022-26474-7

**Published:** 2022-12-19

**Authors:** J. Werth, G. Epro, M. König, A. Santuz, J. Seeley, A. Arampatzis, K. Karamanidis

**Affiliations:** 1 Sport and Exercise Science Research Centre, School of Applied Sciences, London South Bank University, 103 Borough Road, London, SE1 0AA UK; 2 Department of Training and Movement Sciences, Humboldt-Universität zu Berlin, Berlin, Germany; 3 Berlin School of Movement Science, Humboldt-Universität zu Berlin, Berlin, Germany

**Keywords:** Laboratory techniques and procedures, Motor control

## Abstract

This study investigated transfer of improvements in stability recovery performance to novel perturbations. Thirty adults (20-53 yr) were assigned equally to three treadmill walking groups: groups exposed to eight trip perturbations of either low or high magnitude and a third control group that walked unperturbed. Following treadmill walking, participants were exposed to stability loss from a forward-inclined position (lean-and-release) and an overground trip. Lower limb joint kinematics for the swing phase of recovery steps was compared for the three tasks using statistical parametric mapping and recovery performance was analysed by margin of stability and base of support. The perturbation groups improved stability (greater margin of stability) over the eight gait perturbations. There was no group effect for stability recovery in lean-and-release. For the overground trip, both perturbation groups showed similar enhanced stability recovery (margin of stability and base of support) compared to controls. Differences in joint angle kinematics between treadmill-perturbation and lean-and-release were more prolonged and greater than between the two gait perturbation tasks. This study indicates that: (i) practising stability control enhances human resilience to novel perturbations; (ii) enhancement is not necessarily dependent on perturbation magnitude; (iii) differences in motor response patterns between tasks may limit transfer.

## Introduction

Human locomotion frequently faces a variety of perturbations to stability that provoke adjustments to maintain postural integrity and avoid falls. It has been suggested that the central nervous system monitors and corrects motor responses based on the prediction of sensory consequences of perturbations^1^. This must, however, be accurate to the nature of perturbation, and motor control is therefore constantly refined based on error-feedback information^2,3^. In mechanical terms, the system uses an internal representation of the centre of mass (CoM) in relation to the base of support (BoS) based on prior experience^4,5^. If exposed to perturbations which lead to excursion of the CoM beyond the boundaries of the BoS (a state of instability^6,7^), such information will be received, and appropriate motor responses follow, to regain the desired state of the CoM, i.e. a stable body configuration. Given such capability of neuromotor processing, recovery responses adapted from exercised exposure to perturbations could enhance coping with altered forms of the exercised perturbation^8–11^. Based on these assumptions, developing stability control through repeatedly perturbing locomotion has been recognised as an important paradigm for acquisition of general skills for resisting falls in daily life^12,13^.

Extensive research studies have attempted to mimic real-life situations of postural threat during locomotion in an exercise context (e.g. trips or slips). These indicate that single sessions comprising repeated perturbations can elicit acute and retainable improvements in stability control in adults across the lifespan^9,14–18^. Transfer of such exercise-induced stability improvements to altered forms of the exercised perturbations has been revealed previously^9,10,16–18^, and reported to reduce falls incidence in the daily lives of community-dwelling adults^19,20^ - although not for all types of falls. Those results are in line with the general assumption that such skill transfer relies on commonality of contextual sensory feedback between exercised and non-exercised perturbations, requiring only that the system fine tunes the adapted motor response^9,11,21^. If the context of perturbation differs, the system needs to adjust its adapted motor response to the novel task constraints to achieve positive transfer. Such might be readily achieved if the different perturbations elicit some degree of shared stability response for recovery. In our three recent studies^15,22,23^ we could not, however, show functionally relevant matching^22^ or performance transfer from repeated treadmill-based gait perturbations^15,23^ to a clinical fall-risk assessment in the form of a lean-and-release task^24^. Performance transfer failed to occur even though both tasks shared the same direction of perturbation (anterior) and the same stability control mechanism (a rapid anterior recovery step after stability loss). We suggested distinctive synergistic control of muscles as a potential factor limiting transfer of motor skill adaptations between the two tasks^23^. Nevertheless, since exercised stability control via repeatedly perturbed locomotion has been confirmed to be beneficial in resistance to non-exercised perturbations, transfer most likely relies on other factors yet to be examined.

One such factor could be the perturbation magnitude, which directly affects the applied motor error during exercise. There is evidence that adaptations in stability control to treadmill-based slip-like perturbations of lower magnitude can be transferred to even higher magnitude perturbations in a situation for which the contextual information of the motor error is the same^11^. Accordingly, exercising at a certain perturbation magnitude could result in motor outputs appropriate to different perturbation magnitudes, potentially even to different perturbation tasks. Another study investigating slip-like perturbations indicated that transfer of adapted stability control requires exposure to exercised perturbation of high magnitude^25^- of higher magnitude than that sufficient to elicit adaptation during exercise^19^. Thus, for the perturbation magnitude used in our treadmill-based perturbation paradigms the given perturbation magnitude might have been too low for provoking transfer, hence it can be suggested that an increased perturbation magnitude during exercise leads to greater adaptations^26^ and elicits performance transfer. In contrast to this, modelling and experimental studies have argued that there is a nonlinear relationship between the sizes of error feedback and of adaptation^27^, suggesting that greater motor errors would neither mean greater adaptation nor greater transfer of motor skill adaptations.

Therefore, in addition to our previous two transfer investigations^15,23^, this study examined whether an increased perturbation magnitude would elicit or even enhance transfer of adapted recovery performance from short-term treadmill-based gait-trip exercise to unpractised stability loss from a static forward-inclined position (a lean-and-release task) and to an overground trip. These transfer tasks share a similar stability control mechanism (increase in the BoS in the anterior direction by stepping) as that required for treadmill-based gait-trips. By including an overground trip, we have broadened investigation of recovery responses and knowledge of transfer between different perturbations. We hypothesised that exposure to higher perturbation magnitudes during exercise would lead to more pronounced transfer to unpractised perturbation tasks.

## Methods

### Participants and experimental design

Thirty healthy and moderately physically active adults (20-53 years of age) were recruited. Potential participants were excluded if they had any neurological or musculoskeletal injuries or impairments or were over 55 years in age in order to avoid bias caused by problems related to locomotion, whether from natural ageing, disease or trauma. After informed consent was obtained from all participants, they were randomly assigned to three groups of equal size. Two groups were exposed to eight successive trip-like perturbations while walking on a treadmill, with perturbations applied at higher perturbation magnitude or lower magnitude (TRM_high_ group_,_ *n* = 10, three females, averages and standard deviations of age, body height and body mass: 25 ± 5 yr, 1.77 ± 0.77 m, 81.1 ± 16.6 kg; TRM_low_ group, *n* = 10, four females, 29 ± 9 yr, 1.75 ± 0.17 m, 70.9 ± 13.7 kg). A control group walked unperturbed on the treadmill for a similar duration (approximately 20 min) to the other groups (CTR group, *n* = 10, one female, 33 ± 10 yr, 1.80 ± 0.84 m, 82.7 ± 13.8 kg). Afterwards, a single trial of each of two non-exercised transfer tasks took place in the same order for all participants, i.e. at first stability recovery after sudden release from a static forward-inclined position (lean-and-release task), followed by stability recovery after a trip-like perturbation while walking over a flat surface (overground trip). There were short rests of 10 min between each task. Participants wore their own non-slippery leisure/sports shoes throughout all measurements. They were protected by wearing a safety harness connected to an overhead track that allowed for full range of motion in anterior-posterior and medio-lateral directions but prevented contact of any part of the body with the ground (except for the feet). Measurements were reviewed and approved by the ethics committee of the School of Applied Sciences at London South Bank University (approval ID: SAS1826b) and met all requirements for human experimentation in accordance with the Declaration of Helsinki^28^.

### Trip-like perturbation exercise

**Figure 1:**
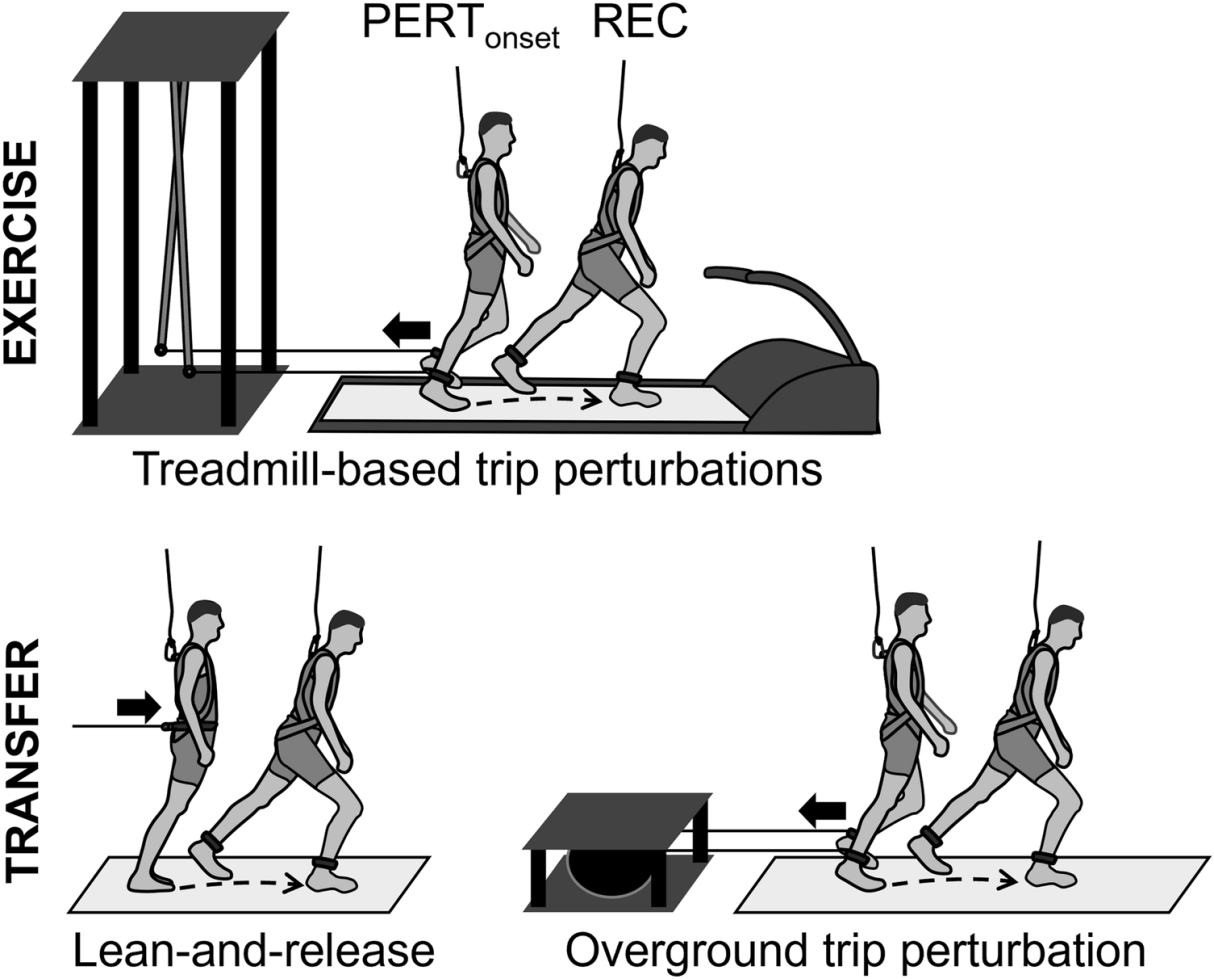
Schematic illustration of the exercised and the two transfer perturbation tasks. The **exercised task** consisted of eight successive trip-like gait perturbations on a treadmill. Perturbations were induced manually using a custom-built electronically driven pneumatic cylinder system at unexpected times during a swing phase of the left leg (PERT_onset_) causing subsequent touchdown (PERT) followed by a recovery step with the right leg (REC). In the **first transfer task** after treadmill-based exercise (Lean-and-release), participants were released from a forward-inclined position once only. The lean angle was normalised to for each participant (33% of body weight). In the **second transfer task**, which followed lean-and-release (Overground trip perturbation), participants were exposed to one trip-like overground gait perturbation (gait speed matched to treadmill speed at 1.4 m·s^-1^) induced manually using a method as for treadmill-based trip-perturbations. Safety harnesses were worn for all trials to prevent contact of any part of the body with the ground (except for the feet).

The trip-like perturbation paradigm has been used in previous studies^14,15,29,30^. Four to seven days prior to measurements, all participants were familiarised with unperturbed treadmill walking. Participants walked on a treadmill (Valiant 2 sport XL; Lode B.V., Groningen, The Netherlands) at a standard speed (1.4 m∙s^−1^). A Teflon cable and ankle strap were connected from both of a participant’s ankles to a custom-built pneumatically driven perturbation device located behind the treadmill (Fig. 1). The strap created a negligible resistance of less than 3 N. Following four minutes of walking^31^, recordings of twelve consecutive steps served to determine stability control during unperturbed walking^29^. As the participants continued to walk, eight trip-like perturbations were induced unexpectedly, with each successive perturbation being followed by variable washout periods (2-3 min) of unperturbed walking^29,30^. The perturbations were induced by activating a pneumatic cylinder using a hand trigger connected to the perturbation device. A restraining force was thereby applied to the left limb via a Teflon cable and ankle strap during mid-stance phase of the right foot to standardise an interruption to motion of the left limb during its mid-swing (i.e. anterior velocity of the lateral malleolus equalled zero). The restraining force was released at touchdown of the left foot to allow for continuity in walking after the perturbation. The subsequent anterior increase in the BoS using the contralateral right leg was defined as the recovery step. One group (TRM_low_) was perturbed in a manner that has previously been shown to improve retainable fall-resisting skills (100 N restraining force, rise time ~ 20 ms^14^). Another group (TRM_high_) was exposed to an increased perturbation magnitude (140 N, rise time ~ 20 ms). Although participants were informed of being perturbed at some points during walking and were encouraged to continue walking, the onset and removal of the resistance was applied without any immediate warning. The CTR group walked without straps and unperturbed at the same standard speed (1.4 m∙s^-1^) for a similar period of time as the perturbation groups.

### Lean-and-release transfer task

This task was operated according to previous studies^32–34^. Participants were forward-inclined with their feet placed flat and at hip-width on the first of two force platforms mounted in series (1080 Hz, 60 × 90 cm, Kistler, Winterthur, Switzerland; Fig. 1). The inclination was maintained by means of an inextensible, horizontally running supporting cable attached to a belt around the participant’s pelvis and at the other end to a custom-built pneumatically driven brake-and-release system. The inclination was set with 33 ± 2% of participant body mass as measured by a load cell incorporated into the supporting cable^32,33^. During task instruction participants were asked to choose the right leg to recover stability after release using a single step onto the second force platform. To initiate the perturbation the supporting cable was suddenly released within 10-30 s after the participant was stabilised in the starting position. We decided not to determine the maximal lean angle from which a participant is still able to recover with a single forward step via multiple exposures to stability loss^34^, to mitigate bias on transfer by potential task adaptation and ensure unpractised task condition. Accordingly, no prior practice trials were used for this task.

### Overground trip transfer task

Participants walked at a standard speed (1.4 m∙s^−1^) on a custom-built flat wooden walkway (8 m length, 1.2 m width), with Teflon cables and ankle straps attached to both ankles^35^. The cables were in turn attached to a custom-built pneumatically driven brake-and-release device located behind the walkway (Fig. 1). Walking speed was monitored live via an optical motion capture system that recorded a reflective marker located on the seventh cervical vertebra (16 infrared cameras operating at 120 Hz; Miqus2, Qualisys, Gothenburg, Sweden). Once participants arrived at the end of the walkway, they were guided back to the initial position to prevent tangling of the Teflon cable. Thus, only one direction was considered for measurements. Following familiarisation with this walking, recordings of three consecutive forward walking trials (in total 12 steps as for the *Trip-like perturbation exercise*) served to determine stability control during movement on the walkway. Subsequently, a single trip-like perturbation was induced randomly within the subsequent five to ten forward-walking trials, when the standard speed was consistently reached^35^. As for *Trip-like perturbation exercise*, the perturbation was operated by means of a hand trigger connected to the perturbation device and evoked by a braking action of the Teflon cable on the left leg—during the mid-stance phase of the right foot and released at touchdown of the left foot. Note that the strap created a negligible resistance of less than 3 N during unperturbed walking. The subsequent anterior increase in the BoS using the contralateral right leg was defined as the recovery step. Although participants were informed that their walking would be perturbed at some point and they were encouraged to continue walking after perturbation, the onset and removal of the resistance was applied without any immediate warning. Similar to the *Lean-and-release transfer task* there were no prior practice perturbation trials, ensuring an unpractised task condition.

### Data collection and processing

In addition to the marker on the seventh cervical vertebra, a further eight reflective markers were tracked via the optical motion capture system. These were placed on both greater trochanters, lateral epicondyles of the femur, lateral malleoli, and the tips of the big toes. Three-dimensional coordinates of the markers were smoothed using a fourth-order digital Butterworth filter with a cut-off frequency of 20 Hz. To assess the state of stability, the anteroposterior margin of stability (MoS) was calculated in accordance with Hof and colleagues^36^ as the difference between the extrapolated CoM (X_CoM_) in the anterior direction and the anterior boundary of the BoS (front toe marker at foot touchdown). The X_CoM_ was calculated based on^37^ as follows:

$${\text{X}}_{CoM} = { }P_{CoM + } \frac{{\frac{1}{2}\left( {V_{CoM} + V_{C7} } \right) + \left| {V_{BoS} } \right|}}{{\sqrt{\frac{g}{L}} }};$$

with: P_CoM_ the anteroposterior component of the vertical projection of the CoM (average of left and right trochanter markers) to the ground; V_CoM_ the anteroposterior velocity of the CoM; V_C7_ the anteroposterior velocity of the C7 marker; V_BoS_ the anteroposterior velocity of the BoS (calculated from the average velocity of the toe markers during the stance-phase of unperturbed treadmill walking); g the gravitational acceleration; and L the reference leg length (defined by the distance between the right trochanter and the centre of the right lateral malleolus). The BoS was defined as the distance between the anterior boundaries of the leading and trailing feet (i.e., the difference between the projections of the two toe markers). The methods for determination of foot touchdowns during locomotion differed depending on the motor task analysed. For treadmill walking, impact peaks of two 2D accelerometers (1080 Hz; ADXL250; Analog Devices, Norwood, MA, USA) placed over the tibia of each leg were used^38^ as the used treadmill did not incorporate force plates. For the lean-and-release task, touchdown was determined via force platform data, defined by the time at which the vertical ground reaction force exceeded 20 N^32^. During the overground task, the vertical position and acceleration of the heel and toe markers were employed to determine touchdown^39^. Foot toe-off was estimated using the local maximum in the vertical acceleration of the toe marker in relation to its minimum vertical position^39^ for all tasks.

During unperturbed walking on both treadmill and overground, stability was determined as the averaged MoS and BoS across six consecutive foot touchdowns of both legs. The state of instability measured with MoS and BoS at the time of perturbation during both types of walking task was identified at touchdown of the perturbed left foot after resistance was applied (PERT). In the lean-and-release task the state of instability was determined at the release of the supporting cable (i.e. PERT when 50% reduction in the leaning force recorded by the incorporated load cell). Stability recovery performance measured with MoS and BoS was evaluated at foot touchdown of the recovery step with the right leg (REC) for each task. In addition, sagittal joint angles at the ankle, knee and hip were calculated for the swing phase of the recovery step (take-off until touchdown, normalised to 101 points for each task and participant). Subsequent analysis of kinematics served to further examine generalisation of motor output in recovery for the several stability perturbations, which allowed critical examination of the initial assumption that performance transfer would be possible if recovery characteristics (i.e. increase in BoS by stepping) were similar. Accordingly, data from the eighth trials of treadmill-based perturbations, as well as from unique trials of both lean-and-release and overground trip, were used.

### Statistics

Parametric assumptions for both parameters (MoS, BoS) were checked and confirmed using Shapiro-Wilk tests (*P* > 0.05). Possible differences between exercise groups and controls in age, body mass and body height were examined using separate one-factor analyses of variance (ANOVAs). For the treadmill task, only the first (Trial 1) and eighth (Trial 8) perturbations were considered for the analysis of adaptive changes in stability control, as these represent novel and most-practised performances. A two-factor ANOVA was used to analyse potential differences in the duration of perturbation (interruption limb until touchdown left foot), with factors event (levels: Trial 1, Trial 8) and group (levels: TRM_low_, TRM_high_). To assess the effect of perturbation magnitude on stability, two-factor ANOVAs were computed for both MoS and BoS separately, and for Trials 1 and 8, with factors event (levels: unperturbed walking, PERT) and group (levels: TRM_low_, TRM_high_). In addition, two-factor ANOVAs, with factors event (levels: unperturbed walking, REC) and group (levels: TRM_low_, TRM_high_), were computed for the BoS, separately for Trials 1 and 8, serving to assess changes in treadmill walking in response to the sudden perturbations. Adaptive changes due to exercise were assessed using separate two-factor ANOVAs with factors trial (levels: Trial 1, Trial 8) and group (levels: TRM_low_, TRM_high_) for both MoS and BoS at PERT and REC.

Potential transfer of stability control adaptations from perturbation exercise to performance in the lean-and-release task and overground trip was assessed by comparing MoS and BoS at PERT and REC for the three groups (TRM_low_, TRM_high_ and CTR) using separate one-factor ANOVAs. To compare the duration of perturbation between the eighth treadmill trip and the overground trip, a two-factor ANOVA with factors event (levels: Trial 8, overground trip) and group (levels: TRM_low_, TRM_high_) was conducted. An additional one-factor ANOVA was performed to compare the duration of perturbation during the overground trip for TRM_low_, TRM_high_ and CTR. To evaluate the effect of exposure to an overground trip on stability, two-factor ANOVAs with factors event (levels: unperturbed walking, PERT) and group (levels: TRM_low_ vs. TRM_high_ vs. CTR) were used separately for MoS and BoS. In addition, a two-factor ANOVA with factors event (levels: unperturbed walking, REC) and group (levels: TRM_low_, TRM_high_, CTR) was computed for BoS to assess changes in overground locomotion. Further to the stability analyses for evaluation of performance transfer between tasks, sagittal plane kinematics of ankle, knee and hip joint angles for the recovery step were compared using statistical parametric mapping (SPM) open-source code SPM1d (version M.0.4.8, www.spm1d.org). An incorporated one-factor repeated measures ANOVA from the three tasks (eighth treadmill perturbation, lean-and-release, overground trip) was applied to the kinematic data for each of the three joints. Thereby, a statistical parametric map SPM(*F*) was created by calculating the conventional univariate *F*-statistic at each point of the entire swing phase of the recovery step. If SPM(*F*) crossed a threshold corresponding to 0.99, *post-hoc* SPM(*t*) maps were calculated for each of the three pairwise comparisons. When the SPM(*t*) map crossed the critical threshold, a significant difference (α = 0.01) was found between the pair of trials examined. In cases of significant main or interaction effects, Bonferroni *post-hoc* corrections were applied. Furthermore, for the entire swing phase of the recovery steps, root mean square errors (RMSE) were computed for the three joints (°) to determine the averaged difference in absolute magnitude observed for lean-and-release task as well as for overground trip kinematics from those during the eighth treadmill-based trip. All analyses were performed using SPSS Statistics (v27, IBM; Chicago, IL, USA) and MATLAB (2020b, MathWorks®, Natick, MA, USA) and, if not stated otherwise, statistical significance was set at *α* = 0.05. Data in results and Figures are given as averages ± standard deviations.

## Results

### Stability control for treadmill-based perturbation exercise

**Figure 2:**
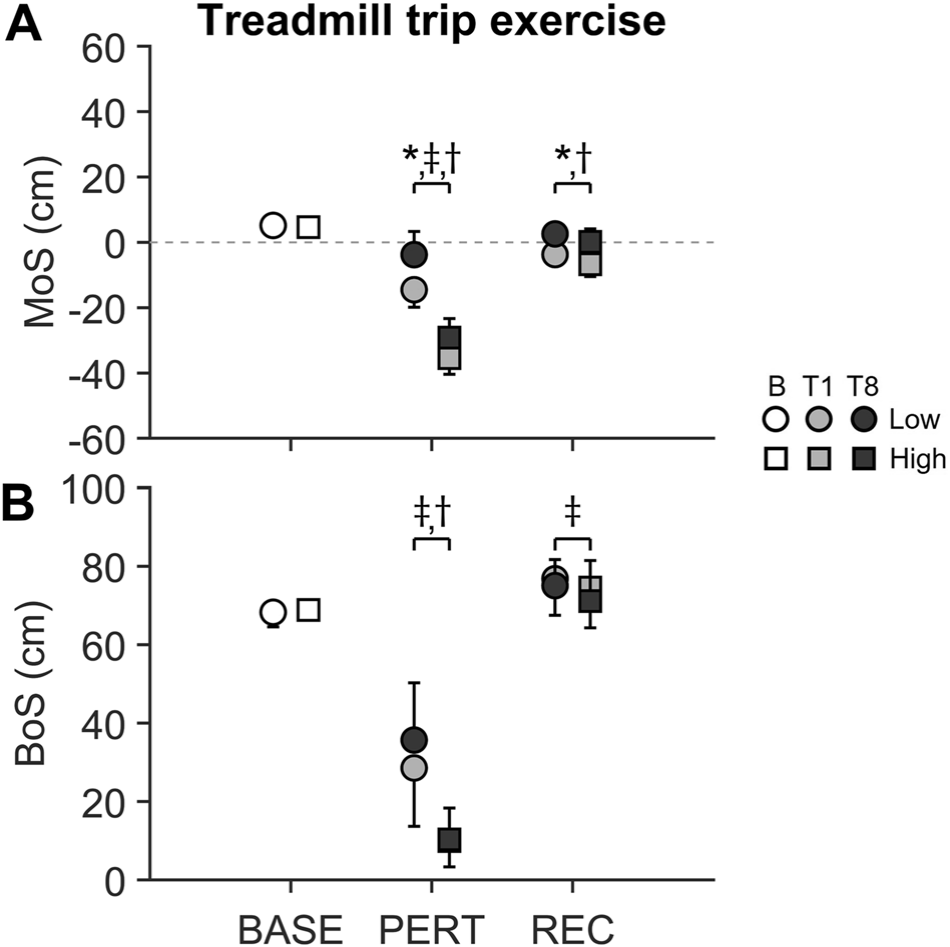
Margin of stability (MoS, **A**) and base of support (BoS, **B**) for unperturbed baseline walking (B, BASE) and for the first (T1) and eighth (T8) trials of treadmill-based perturbation exercise for Low (*n* = 10) and High (*n* = 10) perturbation magnitude groups. Data for T1 and T8 is shown for left foot touchdown after perturbation (PERT) and for the right foot touchdown for the subsequent recovery step (REC). Note that PERT at T1 and T8 (9.9 ± 6.9 cm and 10.4 ± 8.5 cm respectively) showed quite similar average values for the high perturbation magnitude group. Values are presented as averages with standard deviation error bars. ‡, * and † indicate significant differences. ‡ BASE vs. T1 and T8 for low and high groups (*P* < 0.05); * T1 vs. T8 for low and high groups (*P* < 0.001); † low vs. high groups at T1 and T8 (*P* < 0.05).

The duration of perturbation (interruption limb until touchdown left foot) did not significantly differ between the two perturbation groups, either at Trial 1 (TRM_low_ 193 ± 91 ms vs. TRM_high_ 194 ± 137 ms) or at Trial 8 (121 ± 28 ms vs. 137 ± 41 ms), with differences between trials caused by adaptation, i.e. independent of perturbation magnitude participants contacted the treadmill belt in a shorter time post onset of Trial 8 as opposed to Trial 1 [*F*(1,18) = 16.44, *P* = 0.001, $\eta_{p}^{2}$ = 0.477]. During Trial 1 as well as Trial 8 there was a significantly lower MoS at PERT compared to unperturbed walking in both exercise groups [Trial 1: *F*(1,18) = 733.52, *P* < 0.001, $\eta_{p}^{2}$ = 0.976; Trial 8: *F*(1,18) = 176.16, *P* < 0.001, $\eta_{p}^{2}$ = 0.903]. For both trials, there was an interaction effect by group [Trial 1: *F*(1,18) = 86.49, *P* < 0.001, $\eta_{p}^{2}$ = 0.828; Trial 8: *F*(1,18) = 56.31, *P* < 0.001, $\eta_{p}^{2}$ = 0.758]. TRM_high_ compared to TRM_low_ showed a 2.4-fold lower MoS at PERT during Trial (*P* < 0.001; Fig. 2), and a 7.8-fold lower MoS at PERT during Trial 8 (*P* < 0.001; Fig. 2). The BoS at PERT compared to unperturbed walking was lower for both trials in both exercise groups [Trial 1: *F*(1,18) = 391.39, *P* < 0.001, $\eta_{p}^{2}$ = 0.956; Trial 8: *F*(1,18) = 290.77, *P* < 0.001, $\eta_{p}^{2}$ = 0.942]. For both trials, there was an interaction effect by group [Trial 1: *F*(1,18) = 14.97, *P* = 0.001, $\eta_{p}^{2}$ = 0.454; Trial 8: *F*(1,18) = 23.04, *P* < 0.001, $\eta_{p}^{2}$ = 0.561]. TRM_high_ compared to TRM_low_ showed a 2.9-fold lower BoS at PERT during Trial 1 (*P* = 0.003; Fig. 2), and a 3.6-fold lower BoS at PERT during Trial 8 (*P* < 0.001; Fig. 2). During Trial 8, both perturbation groups showed a more positive MoS on average (more stable state of stability) at PERT compared to Trial 1 [*F*(1,18) = 20.79, *P* < 0.001, $\eta_{p}^{2}$ = 0.536; Fig. 2]. The MoS at REC during Trial 8 was higher than during Trial 1 for both exercise groups [*F*(1,18) = 62.03, *P* < 0.001, $\eta_{p}^{2}$ = 0.775; Trial 8 of 1.3 ± 3.4 cm vs. Trial 1 of -5.3 ± 3.3 cm], with an overall slightly lower MoS for TRM_high_ compared to TRM_low_ [Trial 1 and Trial 8; *F*(1,18) = 4.17, *P* = 0.044, $\eta_{p}^{2}$ = 0.348]. Whilst there were no differences between exercise groups, nor between perturbation trials (Trial 1 vs. Trial 8), the BoS at REC was higher compared to unperturbed walking during both perturbation trials [Trial 1: *F*(1,18) = 7.23, *P* = 0.015, $\eta_{p}^{2}$ = 0.286; Trial 8: *F*(1,18) = 5.92, *P* = 0.026, $\eta_{p}^{2}$ = 0.248].

### Transfer of exercised stability control to novel perturbations

**Figure 3:**
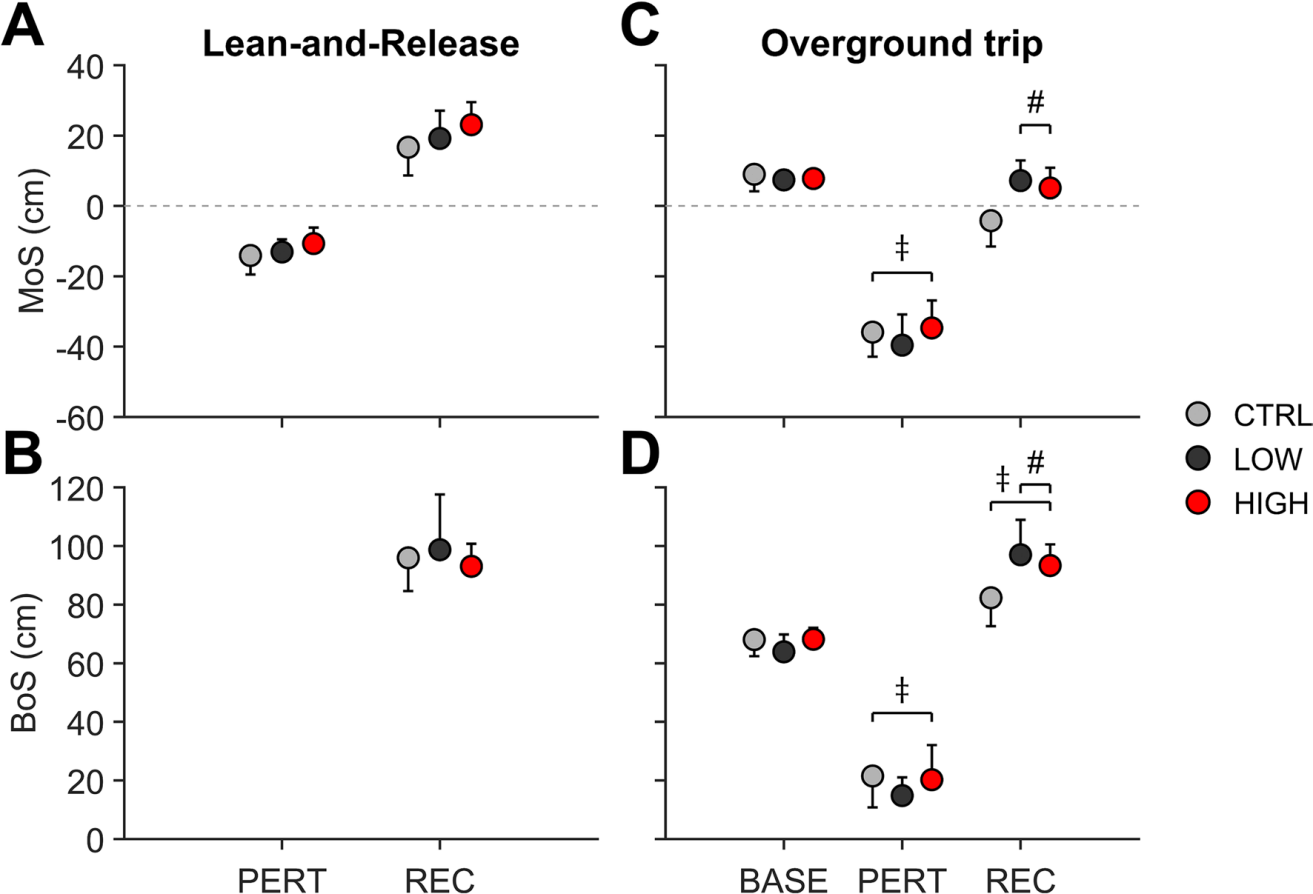
Margin of stability (MoS, **A** and **C**) and base of support (BoS, **B** and **D**) for the Lean-and-release and Overground trip transfer tasks for low (*n* = 10) and high (*n* = 10) perturbation magnitude groups and controls (*n* = 10; CTR). Data is shown for cable release or left foot touchdown after gait perturbation (PERT) and subsequent right foot touchdown for recovery step (REC) for all groups and unperturbed baseline walking (BASE) only for the overground task. Note that the BoS at PERT for lean-and-release equalled zero for all groups and hence is not shown. Values are presented as averages with standard deviation error bars. ‡ and # indicate significant differences. ‡ vs. BASE (*P* < 0.001); # vs. CTR (*P* < 0.05).

There were no significant group effects for age, body height, and body mass between TRM_high_, TRM_low_, and CTR [age, *F*(2,27) = 2.46, *P* = 0.105, $\eta^{2}$ = 0.154; body height, *F*(2,27) = 0.51, *P* = 0.610, $\eta^{2}$ = 0.042; body mass, *F*(2,27) = 1.73, *P* = 0.200, $\eta^{2}$ = 0.131]. The state of instability caused by the perturbation in the lean-and-release task (MoS at release of the supporting cable, i.e. PERT) did not differ amongst the three groups [TRM_high_, TRM_low_, CTR; *F*(2, 27) = 1.47, *P* = 0.248, $\eta^{2}$ = 0.098; Fig. 3]. Participants of all groups were able to recover stability within a single step. The analysis of potential treadmill-based transfer in stability performance showed no significant effects, neither between the exercise groups nor between them and the control group [for MoS and BoS at REC: *F*(2,27) = 1.86, *P* = 0.175, $\eta^{2}$ = 0.121 and *F*(2,27) = 0.48, *P* = 0.623, $\eta^{2}$ = 0.034; Fig. 3].

With respect to the overground trip, the duration of perturbation did not significantly differ amongst the groups (TRM_low_ 129 ± 43 ms; TRM_high_ 118 ± 34 ms; CTR 128 ± 44 ms), nor differed in duration for either exercise group compared to Trial 8 of treadmill-based exercise. The overground trip perturbation caused a lower MoS at PERT compared to unperturbed walking [*F*(1,2) = 822.14, *P* < 0.001, $\eta_{p}^{2}$ = 0.968], with no significant differences amongst the three groups for either of the two events (Fig. 3). Furthermore, the BoS at PERT compared to unperturbed walking was lower [*F*(1,2) = 549.37, *P* < 0.001, $\eta_{p}^{2}$ = 0.953] in all three groups, with no significant group effects at neither of the two events (Fig. 3). However, there was a significant group effect for REC [*F*(2,27) = 9.24, *P* < 0.001, $\eta^{2}$ = 0.406]. *Post-hoc* tests showed a significantly higher MoS for both TRM_low_ (*P* = 0.001) and TRM_high_ (*P* = 0.008) compared to CTR, with no effect of the exercised perturbation magnitude (TRM_low_ vs. TRM_high_) on transfer performance (Fig. 3). Note that all participants used a lowering strategy to recover from the overground gait perturbation, i.e. anterior displacement of the BoS by means of an anterior step of the right leg after touchdown of the perturbed left foot (as it was evoked during treadmill-based exercise). Whilst all groups showed a significantly higher BoS at REC compared to unperturbed walking [*F*(1,2) = 153.20, *P* < 0.001, $\eta_{p}^{2}$ = 0.850], there was yet further a group effect [*F*(2,27) = 6.35, *P* = 0.005, $\eta^{2}$ = 0.320]. *Post-hoc* tests indicated significance for TRM_low_ (*P* = 0.006) and for TRM_high_ (*P* = 0.048) for the BoS at REC compared to CTR.

**Figure 4:**
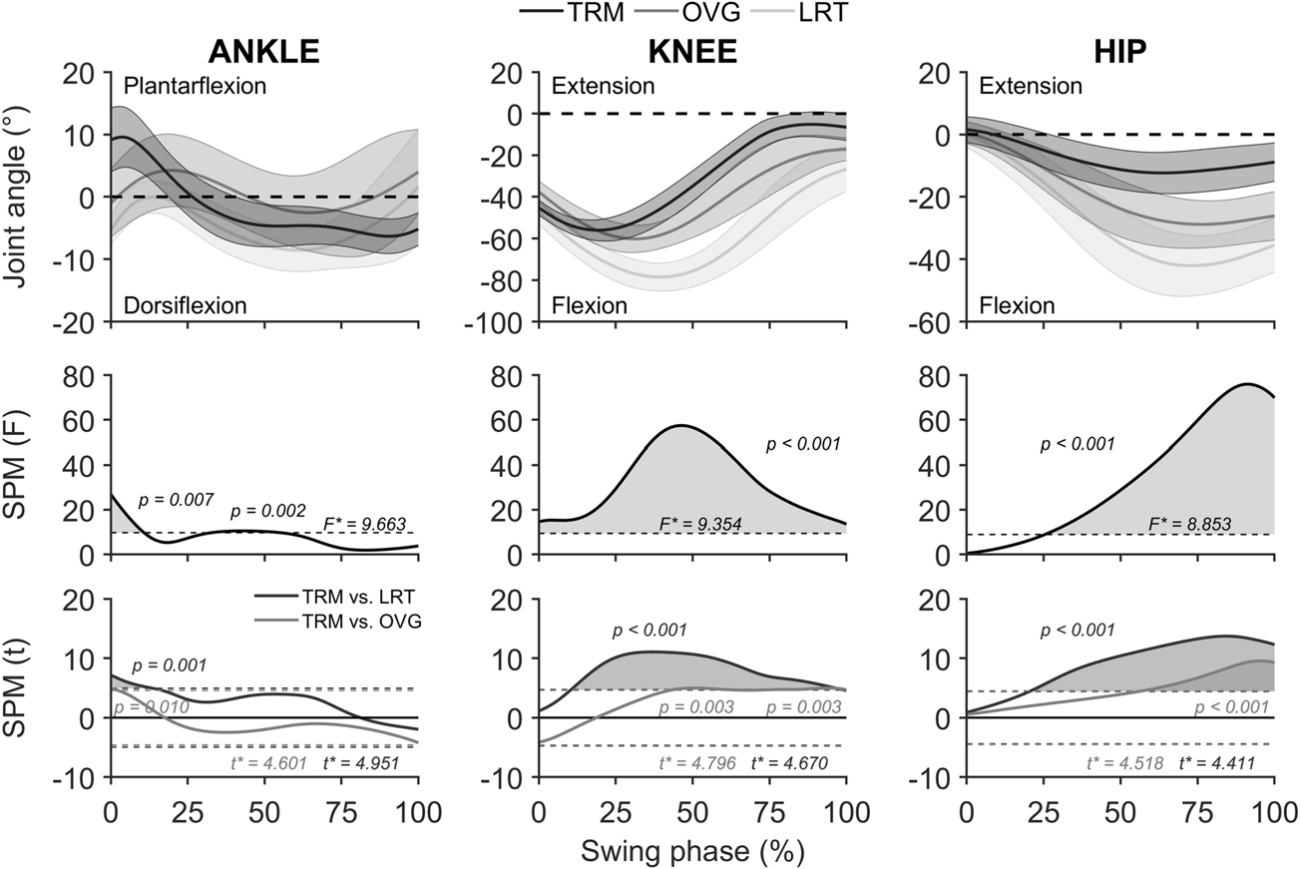
Sagittal plane joint angle kinematics for the low perturbation magnitude group (*n* = 10) and statistical parametric mapping (SPM) analyses of the ankle, knee, and hip joint. Graphs show the entire swing phase of the recovery step (toe-off to touchdown, 0-100%) for the eighth treadmill-based trip (TRM), overground trip (OVG) and lean-and-release (LRT). **First row**: Joint angle comparisons for all three tasks (bold lines as averages and shaded areas as standard deviations). **Second row**: SPM one-way repeated measures ANOVA [SPM(*F*)] and univariate *F*-statistic (F*) with significance threshold at 99% confidence (dashed line) with task as factor (TRM, OVG and LRT). The shaded grey areas indicate significant amongst the three tasks. **Third row**: post-hoc tests [SPM(*t*)] comparing pairs of joint angle curves (i.e. TRM vs. LRT in black and TRM vs. OVG in grey). *t*-statistic (t*) with significance threshold at 99% confidence (dashed lines) and shaded areas indicating significant differences between the pairs of tasks.

The durations of the swing phases for recovery steps were as follows: for the 8^th^ treadmill trips, 328 ± 50 ms, 208 ± 53 ms; for lean-and-release, 235 ± 53 ms, 234 ± 33 ms; for the overground trip, 198 ± 33 ms, 169 ± 25 ms (TRM_low_ and TRM_high_ respectively throughout). In relative terms, SPM analyses for the TRM_low_ group [SPM(*F*); Fig. 4] computed across the three perturbation tasks (eighth treadmill trip vs. lean-and-release vs. overground trip) but separately for each joint showed significant main effects for ankle (0-11% of swing phase, *P* = 0.007; 33-55%, *P* = 0.002), knee (0-100%, *P* < 0.001) and hip (25-100%, *P* < 0.001). *Post-hoc* tests [SPM(*t*), Fig. 4] on the knee and hip joint angle kinematics revealed that the lean-and-release task compared to the treadmill trip was recovered with significantly more knee as well as hip flexion for most of the swing phase (knee, 10-99%, *P* < 0.001; hip, 20-100%, *P* < 0.001). Furthermore, recovery from the lean-and-release task compared to the treadmill trip involved significantly higher dorsiflexion (*P* = 0.001) over 0-14% of the swing phase. In comparison to this, the overground trip compared to the treadmill trip differed only for 27% of the swing phase in knee (45–59% and 86–99%, *P* = 0.003), 43% in hip (57-100%, *P* < 0.001) and 2% ankle joint (0-2%, *P* = 0.010).

**Figure 5:**
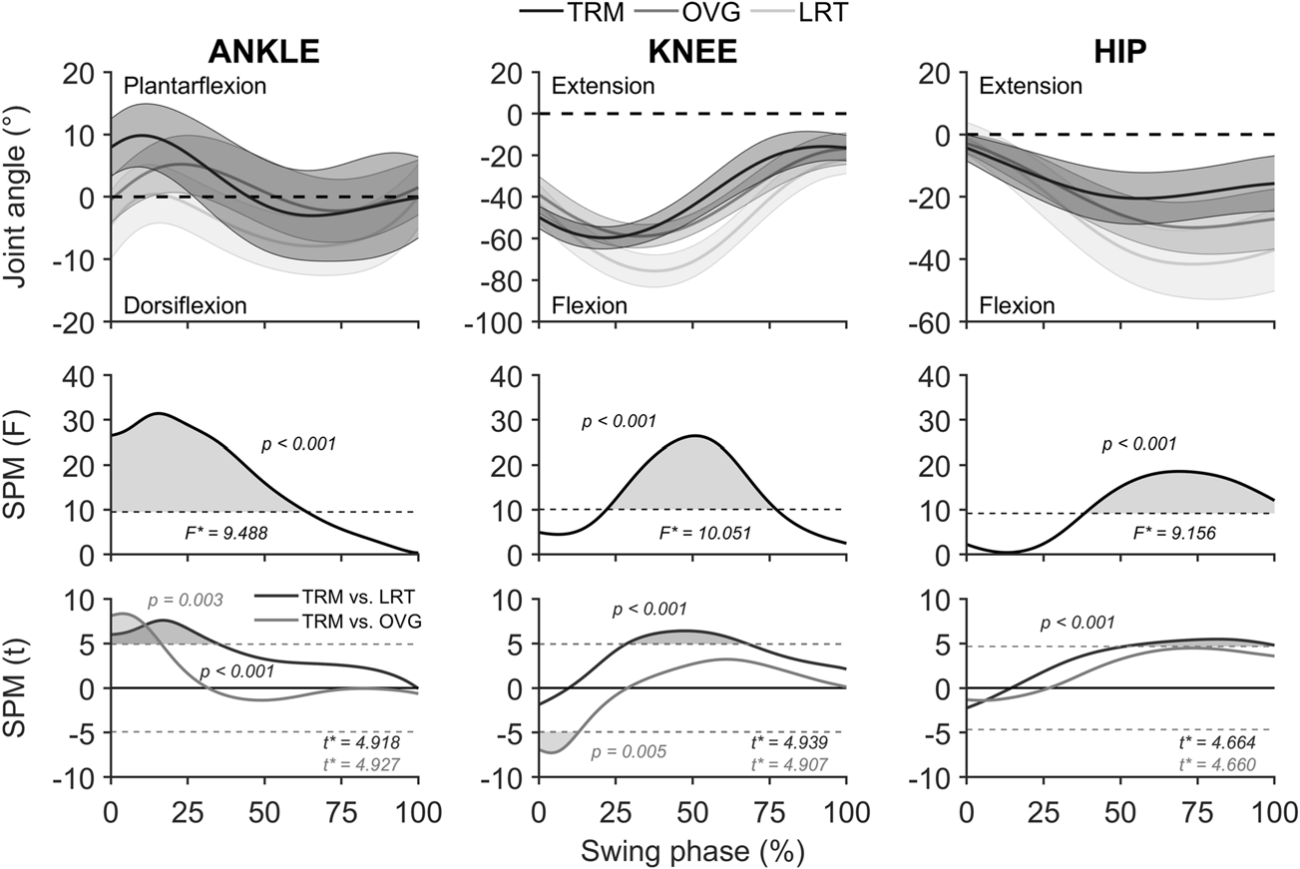
Sagittal plane joint angle kinematics for the high perturbation magnitude group (*n* = 10) and statistical parametric mapping (SPM) analyses of the ankle, knee, and hip joint. Graphs show the entire swing phase of the recovery step (toe-off to touchdown, 0-100%) for the eighth treadmill-based trip (TRM), overground trip (OVG) and lean-and-release (LRT). **First row**: Joint angle comparisons for all three tasks (bold lines as averages and shaded areas as standard deviations). **Second row**: SPM one-way repeated measures ANOVA [SPM(*F*)] and univariate *F*-statistic (F*) with significance threshold at 99% confidence (dashed line) with task as factor (TRM, OVG and LRT). The shaded areas indicate significant amongst the three tasks. **Third row**: post-hoc tests [SPM(*t*)] comparing pairs of joint angle curves (i.e. TRM vs. LRT in black and TRM vs. OVG in grey). *t*-statistic (t*) with significance threshold at 99% confidence (dashed lines) and shaded areas indicating significant differences between the pairs of tasks.

A similar trend for inter-task differences in kinematics was found for the TRM_high_ group (Fig. 5). Recovery from the lean-and-release task compared to the treadmill task differed in total for 40% of the entire swing phase in knee (28-68%, *P* < 0.001), 49% in hip (51-100%, *P* < 0.001), and 35% in ankle (0-35%, *P* < 0.001) joints. In contrast, overground compared to treadmill trip showed only differences for the knee and the ankle joints at the initiation of the swing phase (ankle 0-13%, *P* = 0.003; knee 0-17%, *P* = 0.005; Fig. 5). Furthermore, the overground trip compared to the lean-and-release task showed ~ 1.5- to twofold lower differences in absolute size in knee and hip joint angle kinematics for the swing phase of the recovery step from the treadmill trip, and that independent of the exercise group [RMSE for the overground trip vs. the lean-and-release task compared to the treadmill trip in knee (TRM_low_, 15 vs. 30° and TRM_high_, 11 vs. 20°) and in hip (TRM_low_, 14 vs. 21° and TRM_high_, 8 vs. 17°].

## Discussion

Factors that elicit or limit transfer of stability control adaptations have not yet been thoroughly investigated. The current study tested transfer potential for an established perturbation magnitude (MoS_low_) previously shown as being sufficient to elicit acute refinements of motor responses^14,15,29,30^, and further examined the influence of an increased perturbation magnitude (MoS_high_) on fall-resisting skill adaptation performance transfers to other tasks. The general assumption for positive transfer from exercised skills to novel tasks could be confirmed with both exercise groups adapting stability control (skill refinement) from a single session of repeated treadmill-based trip perturbations. Moreover, recovery performance to a non-exercised overground trip after treadmill-based exercise was enhanced compared to controls indicating that treadmill-based perturbation paradigms have the potential to mitigate fall risk during overground tripping. Concerning the TRM_low_ group, it is worth noting that even though the state of instability observed for treadmill-based gait perturbations was 2.7-fold lower compared to the novel overground trip (average MoS at PERT −14.5 cm vs. −39.6 cm), participants showed enhanced stability recovery performance after overground tripping compared to the control group. These findings match previous evidence of transfer of adaptations from exercising with lower to higher magnitudes of treadmill-based slip-like perturbations^11^. It may therefore be suggested that the central nervous system is capable of rapidly calibrating the required motor action to cope with higher magnitude of perturbation purely based on information from prior exposure of lower magnitude^40^, i.e. repeated increase in BoS.

Contrary to our extended hypothesis, an increase in exercise perturbation magnitude did not lead to enhanced transfer performance in an overground trip, confirming that greater motor errors do not necessarily lead to greater adaptation or inter-task transfer of adaptative changes. As expected, an increased perturbation magnitude during exercise led to greater motor errors (lower MoS and BoS in relation to unperturbed walking) during both the first (novel) and eighth treadmill trip trials, indicating a generally higher demand on the neuromotor system for execution of appropriate recovery responses. Interestingly, although the absolute change in MoS as well as in BoS between the perturbation and subsequent recovery step was higher in TRM_high_ compared to TRM_low_, it resulted in only slightly different states of stability at REC across trials (Fig. 2), and in no group differences in BoS at REC. These results suggest that the central nervous system can appropriately calibrate motor responses to the specific perturbation magnitude for the first (non-exercised) perturbation, and only fine tunes the response for subsequent perturbations. The absence of any magnitude effect on MoS or BoS for recovery touchdown, however, does indicate that recovery responses were executed to the minimum required (i.e. positive MoS) to preserve continuity of walking post recovery. Nevertheless, independent of the exercise group, the BoS at REC was higher compared to unperturbed walking during both Trials 1 and 8 of exercise. This highlights that exposure to perturbations per se elicits an increase in BoS higher than for unperturbed walking to control stability, which is potentially a crucial factor for inter-task transfer performance. This would explain the significantly higher BoS at REC after overground perturbation in both exercise groups compared to the control group, contributing to achieve a positive MoS (i.e. control of CoM within the boundaries of the BoS) and hence a stable state after recovering from a threat of falling.

In our previous studies we did not detect any transfer of treadmill-based trip resilience to the recovery performance in a lean-and-release task^15,23^ and provided evidence that this might be explained by differences in the task-specific neuromuscular control of motor output^23^. The current study aimed to test the possibility that variation in perturbation magnitude during treadmill-based exercise (higher than in our previous studies) might alter transfer performance. Since neither of the exercise groups (TRM_low_ nor TRM_high_) differed to controls in recovery stability after release, it can be suggested that a potentially increased excitability of the motor cortex through increase in perturbation magnitude during exercise seems redundant for transfer of trip-resisting skills. Furthermore, reviewing joint kinematic patterns indicated extensive differences of the recovery limb between the eighth treadmill gait trip (the adapted stability performance) and lean-and-release recovery for both low- and high-magnitude perturbation groups. Although both perturbation recoveries started with a flexion in knee and hip joints and were followed by dorsiflexion to allow foot clearance for the swing limb^23,41^, a subsequent knee extension and simultaneous hip extension for both tasks served to further increase step length to establish an upright posture at touchdown. Contrarily, the swing phase for lean-and-release as compared with treadmill showed a later onset of subsequent knee extension, not only in relative but also in absolute terms (lean-and-release, TRM_low_, 99 ± 17 ms; TRM_high_, 92 ± 10 ms vs. treadmill, TRM_low_, 69 ± 11 ms; TRM_high_, 53 ± 16 ms) independent of magnitude group [*F*(1,18) = 53.02, *P* < 0.001, $\eta_{p}^{2}$ = 0.757]. The swing phase was notably characterised by significantly higher flexion of both knee and hip joints for a substantial proportion of the entire recovery step (ranging from ~ 40 to 89% across joints and exercise groups (Figs. 4 and 5). Such differences in kinematic patterns of an anterior step between tasks may be related to the different nature of initialisation of perturbations. Even though there was similar or even lower MoS at time of perturbation (depending on exercise group) between lean-and-release and treadmill trip (indicating a similar or lower state of instability caused by the perturbation), the body is inclined more anteriorly in a lean-and-release task to achieve the initial instability, given that the velocity of the CoM at release is ~ zero. This explains, for the lean-and-release task, both a more dorsiflexed ankle configuration at the beginning of the swing phase caused by the greater initial lean angle and the requirement for higher as well as prolonged knee and hip flexion in order to extend the foot clearance of the swing leg and eventually increase the BoS. Differences in task continuity beyond the touchdown of perturbation recovery might influence recovery steps. Following from the establishment of upright posture for recovery touchdown after perturbation, the preparation for subsequent weight acceptance and push-off phases is crucial for treadmill-based perturbations since continuity in walking is not only desired but required. This would be hindered by a higher hip or knee flexion for treadmill touchdowns as opposed to single-step recovery after lean-and-release to maintain stable stance after touchdown. When considering the different types of gait perturbations (treadmill vs. overground trip), kinematic differences were either absent (i.e. for the knee joint of the high perturbation magnitude group) or occurred for only ~ 2-43% of swing phase (across joints and exercise groups). Joint kinematic differences between treadmill and overground trips were generally lower in absolute magnitude (RMSE across groups and joints averaged up to 15°) as compared with treadmill vs. lean-and-release with values on average up to 30°. Altogether, and in line with the findings of König and colleagues^23^, it is suggested that the extent of differences in task context leads to different temporal frameworks for the single motor response, i.e. the increase in BoS by anterior stepping. Investigation of the time courses of motor responses may therefore be essential for understanding success or failure of transfer of trip resilience from one task to another.

This study has some limitations. Even though the current method to assess MoS has been identified for sensitive detection of various adaptation and retention effects, e.g.^14,15,23,29,30^, we cannot exclude potential errors introduced by the reduced kinematic model to analyse CoM dynamics. However, as we showed significant transfer effects for overground tripping but not for the lean-and-release task via the analysis of the BoS we think that our conclusions are robust and that any systematic error in CoM calculations would not have significantly affected our main findings. With regards to our joint angle analyses, we wish to acknowledge that we did not analyse 3D movements of the whole segments. Thus, despite that recovery responses were predominantly directed anteriorly, we cannot fully consider any potential movements along the longitudinal axis when using the current method. Furthermore, one might argue that the number of participants analysed in the current study was relatively low, affecting the statistical power for the transfer analysis regarding the lean-and-release task. In this regard, we record that we have analysed 82 (intervention) and 67 (control) participants across our current and previous studies^15,23^, and have consistently identified no transfer. We are therefore confident that the conclusion of the current study is robust. Lastly, we cannot exclude that higher number of perturbations over multiple sessions might lead to lean-and-release transfer, nor can we exclude that populations other than that investigated in our study might reveal different outcomes. Nevertheless, our current and previous study designs^15,23^ have enabled us to investigate specific factors that may be crucial to the design and evaluation of longitudinal interventions in clinical applications.

In conclusion, this study confirmed that repeated exposure to treadmill-based gait perturbations leads to rapid adaptive changes in stability recovery performance and demonstrated that a single session of perturbation exercise has the potential to elicit transfer of stability recovery performance to non-exercised overground trip-like perturbations. However, higher perturbation magnitudes do not necessarily influence transfer performance. Transfer of stability recovery mechanisms and performance from one task to another may partly be subject to the degree of similarity in recovery motor responses between perturbations.

## Data Availability

The datasets generated during and/or analysed during the current study are available from the corresponding author on reasonable request.
